# Elevated temperatures cause loss of seed set in common bean (*Phaseolus vulgaris* L.) potentially through the disruption of source-sink relationships

**DOI:** 10.1186/s12864-019-5669-2

**Published:** 2019-04-24

**Authors:** Ali Soltani, Sarathi M. Weraduwage, Thomas D. Sharkey, David B. Lowry

**Affiliations:** 1 Department of Plant Biology, Michigan State University, East Lansing, MI USA; 2 Plant Resilience Institute, Michigan State University, East Lansing, MI USA; 3 MSU-DOE Plant Research Laboratory, Michigan State University, East Lansing, MI USA; 4 Department of Biochemistry and Molecular Biology, Michigan State University, East Lansing, MI USA

**Keywords:** Heat stress, Common bean, Photosynthesis, Transcriptome, Source-sink relationships

## Abstract

**Background:**

Climate change models predict more frequent incidents of heat stress worldwide. This trend will contribute to food insecurity, particularly for some of the most vulnerable regions, by limiting the productivity of crops. Despite its great importance, there is a limited understanding of the underlying mechanisms of variation in heat tolerance within plant species. Common bean, *Phaseolus vulgaris*, is relatively susceptible to heat stress, which is of concern given its critical role in global food security. Here, we evaluated three genotypes of *P. vulgaris* belonging to kidney market class under heat and control conditions. The Sacramento and NY-105 genotypes were previously reported to be heat tolerant, while Redhawk is heat susceptible.

**Results:**

We quantified several morpho-physiological traits for leaves and found that photosynthetic rate, stomatal conductance, and leaf area all increased under elevated temperatures. Leaf area expansion under heat stress was greatest for the most susceptible genotype, Redhawk. To understand gene regulatory responses among the genotypes, total RNA was extracted from the fourth trifoliate leaves for RNA-sequencing. Several genes involved in the protection of PSII (*HSP21*, *ABA4*, and *LHCB4.3*) exhibited increased expression under heat stress, indicating the importance of photoprotection of PSII. Furthermore, expression of the gene *SUT2* was reduced in heat. SUT2 is involved in the phloem loading of sucrose and its distal translocation to sinks. We also detected an almost four-fold reduction in the concentration of free hexoses in heat-treated beans. This reduction was more drastic in the susceptible genotype.

**Conclusions:**

Overall, our data suggests that while moderate heat stress does not negatively affect photosynthesis, it likely interrupts intricate source-sink relationships. These results collectively suggest a physiological mechanism for why pollen fertility and seed set are negatively impacted by elevated temperatures. Identifying the physiological and transcriptome dynamics of bean genotypes in response to heat stress will likely facilitate the development of varieties that can better tolerate a future of elevated temperatures.

**Electronic supplementary material:**

The online version of this article (10.1186/s12864-019-5669-2) contains supplementary material, which is available to authorized users.

## Background

Heat is among the most devastating abiotic stresses negatively impacting crop production and food security worldwide [1]. Every 1 °C increase in seasonal temperature results in a 10–17% reduction in crop yields [2, 3]. With the global mean temperatures set to increase at a pace of 0.2 °C per decade, the impact of heat on worldwide food production will only become more acute [4]. For some regions of the globe, temperatures are increasing even more rapidly and heat waves are expected to increase in intensity and duration [5].

Elevated temperatures negatively affect several crucial physiological processes in plants. One of the effects of elevated temperature is an increased accumulation of reactive oxygen species (ROS), [6] which are harmful to plant membranes, proteins, and other macromolecules. Photosynthesis is also affected by elevated temperatures, with carbon assimilation reduced as a result of a reduction in rubisco activation [7, 8]. Rubisco activase is inhibited at high temperature, leading to heat-induced deactivation of rubisco under moderate heat stress [9, 10]. Further, heat modifies cell membrane characteristics and consequently affects membrane-binding proteins [11–13]. Photosystem II (PSII) is thought to be particularly sensitive to heat stress. However, negative effects of heat stress on net photosynthesis has not been reported in all crops [14]. Besides carbon assimilation, carbon translocation from source to sink tissues has been shown to be interrupted by heat stress [15]. Continuous sucrose transport from source leaves to developing reproductive tissues is crucial for male fertility, seed set, and seed filling [16, 17]. Pressman et al. [18] found that the disruption of sucrose supply and/or its breakdown to hexoses under heat stress results in male sterility and aborted seed set in tomato, a drastic negative impact of this abiotic stress on the carbon balance of a plant.

Several mechanisms to cope with heat have evolved in plants. Elevated temperatures triggers a suite of physiological responses that can ameliorate the deleterious effects of heat. For example, the ROS scavenging machinery is overexpressed, which will convert ROS to less harmful molecules [19]. ROS also serves as a signaling molecule that can activate heat shock factors (HSFs), [20, 21]. HSFs bind to palindromic motifs in the promoter of heat responsive genes, including heat shock proteins (HSPs). HSPs are molecular chaperones that refold and stabilize protein structures under heat stress. Besides adopting these thermo-tolerance strategies, preventative mechanisms have evolved that improve the cooling capacity of the leaves by increasing transpiration rates [22].

In this study, we focused on understanding how heat-tolerant and heat-sensitive varieties of the common bean, *Phaseolus vulgaris*, respond to elevated temperatures. Common bean is an economically important crop that originated and was domesticated in the New World. *P. vulgaris* is separated into two major genepools: Middle American and Andean [23, 24]. Humans domesticated beans from both of these major gene pools independently [25]. Further, three major races have been characterized within the Middle American genepool: Mesoamerica, Durango, and Jalisco. Similarly, there are three races in Andean genepool: Nueva Granada, Chile, and Peru. After the colonization of the New World, Andean beans were introduced to European and African countries [26]. Today, beans are cultivated in many regions of the world. However, being adapted to moderate climates [27], common bean has limited heat tolerance. Seed yield reductions have been reported for temperatures higher than 30 °C during the day or higher than 20 °C at nights [28]. Heat stress-induced yield reductions were described as a result of flower abscissions, development of parthenocarpic pods (pin pods), lower seed set per pod or decreased seed size [28, 29]. The level of heat sensitivity in beans depends strongly on the developmental stage in which plants are exposed to elevated temperatures. Several studies reported that heat stress at pre-fertilization stages are more detrimental to pollen development and/or anther dehiscence [29, 30]. Furthermore, Shonnard and Gepts [31] reported sensitivity of beans to heat stress at pod filling stages in addition to flower bud formation.

Elevated temperatures are predicted to be more frequent, particularly in some Central American and African countries, where Andean genotypes are the main beans in cultivation [32]. Heat and drought stress often impact crops simultaneously in these regions. While irrigation can be used to eliminate water stress, the direct impacts of heat stress cannot be alleviated by any management practice. Development of heat tolerant varieties remains the only practical solution for minimizing the negative effects of elevated temperatures. Developing bean varieties that can better tolerate elevated temperatures is now considered crucial in both Latin America [33] and Africa [34]. Several heat tolerant varieties have been identified and used in breeding pipelines [31, 33, 35, 36]. Understanding the differential physiological responses of heat-sensitive and heat-tolerant Andean genotypes to elevated temperatures is a crucial step toward improving the thermo-tolerance of this economically important crop.

Here, we investigate the transcriptome and physiological responses of three varieties of Andean beans to elevated temperatures. These varieties were selected based on their tolerance (Sacramento and NY-105) or susceptibility (Redhawk) to heat stress. The main objectives of this study were: *i*) to elucidate the effect of heat stress on the leaf morpho-physiology of the bean plants, *ii*) understand the transcriptomic responses of leaves under elevated temperatures, and *iii*) to identify the potential genes/physiological pathways that are involved in heat stress tolerance. Understanding the physiological mechanism(s) of tolerance to elevated temperatures will help the researchers to develop heat tolerant varieties more efficiently.

## Results

### Effect of elevated temperature on leaf morpho-physiology

**Fig. 1:**
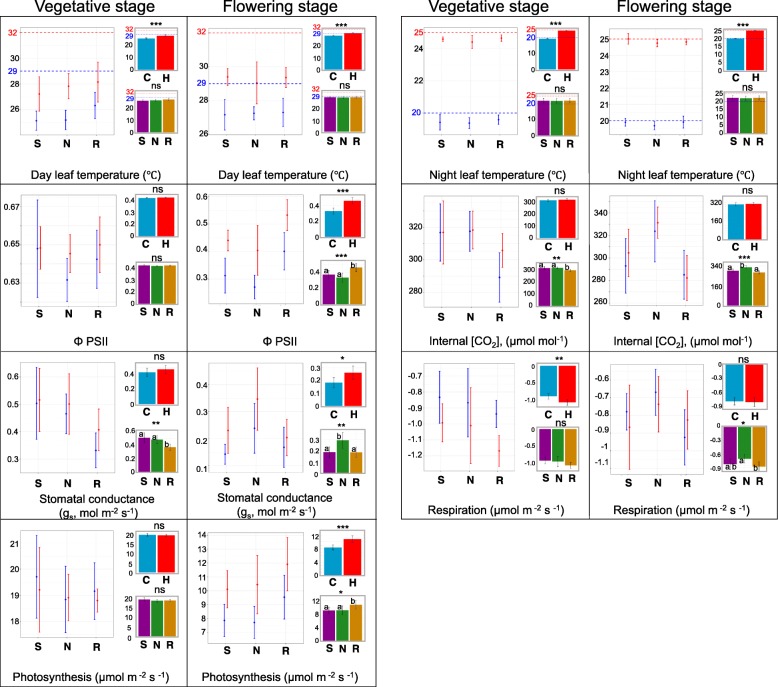
Leaf surface temperatures and photosynthesis characteristics measured for three bean genotypes (Sacramento, NY-105, and Redhawk) grown under control and heat stress treatments. In each figure, the left graph represents the means for each genotype grown under control (blue) or heat (red) conditions. For each pane, the upper right graph represents the main effect of treatments across genotypes while the graph below represents the main effect of genotypes across treatment. The letters on each bar represents the results of post-hoc analysis. The same letter indicates the means are not significantly different at 0.05 probability level. The bars in all figures represent the 95% confidence intervals. In the leaf temperature figures, ambient temperatures in control (blue) and heat (red) stress conditions are highlighted by dashed lines. S = Sacramento, N = NY-105, R = Redhawk. C = control treatment, H = heat treatment

In both vegetative and flowering stages, the leaf temperature at night was similar to the ambient temperatures in both control and elevated temperature conditions (Fig. 1). However, during the day, leaf temperatures were 2-3 °C cooler than the ambient temperatures in both treatment levels.

At the early developmental stage, we did not detect a significant effect of treatment on photosynthetic rate, stomatal conductance, intercellular [CO_2_] (*C*_i_), or ΦPSII. However, plants under elevated temperature conditions had significantly higher dark respiration rates (Fig. 1). At the flowering stage, significant increases were detected in ΦPSII under the elevated temperature (Fig. 1). Similarly, plants grown under elevated temperatures had higher stomatal conductance and photosynthetic rates. The *C*_i_ and respiration rate were similar between the control and elevated temperature treatments for fourth trifoliate at the flowering stage. Among genotypes, Redhawk had the lowest stomatal conductance and the lowest *C*_i_ at the early stage of development. However, Redhawk had the highest ΦPSII and photosynthesis rate across treatments at the later flowering stage. NY-105 had the highest *g*_*s*_ and *C*_i_ at the flowering stage (Fig. 1).

**Fig. 2:**
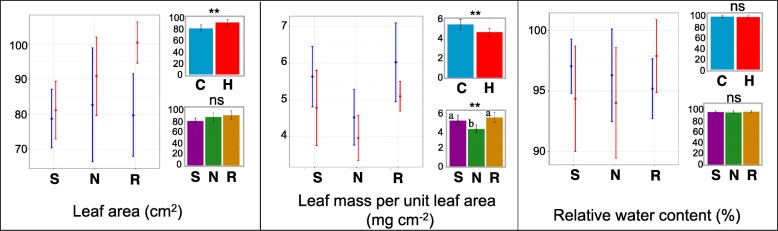
Leaf morphometric traits measured from three bean genotypes (Sacramento, NY-105, and Redhawk) at flowering stage grown under control and heat stress condition. In each figure, the left graph represents the means for each genotype grown under control (blue) or heat (red) condition. The upper right graph represents the main effect of treatments across genotypes and the graph below represents the main effect of genotypes across treatment. The letters on each bar represents the results of post-hoc analysis. The same letter indicates the means are not significantly different at 0.05 probability level. The bars in all figures represent 95% confidence intervals. S = Sacramento, N = NY-105, R = Redhawk. C = control treatment, H = heat treatment

Leaf area was also significantly greater in the elevated temperature treatment at flowering stage (Fig. 2). This increase was most drastic in Redhawk (Fig. 2). In contrast, a significant reduction in leaf mass per unit leaf area was detected for plants in the elevated temperature treatment. Although the main effect of genotype was not significant for leaf area, NY-105 had the lowest leaf mass per unit leaf area (thinnest leaves). Neither treatment nor genotype had a significant effect on relative water content (RWC %), indicating that plants in the elevated temperature treatment were not under water stress (Fig. 2).

Stomatal density was measured for both the abaxial and adaxial sides of leaves at flowering stage (Additional file 7: Figure S2). A significant decrease in stomata density on the abaxial side of leaves was found for plants grown in the elevated temperature treatment. Among genotypes, Sacramento had the highest stomatal density for both adaxial and abaxial sides across treatments.

### Effect of elevated temperature on seed set

A drastic reduction in the number of filled pods, seeds per pod, and total number of plump seeds were detected in plants grown under elevated temperatures (Additional file 8: Figure S3). The heat susceptible genotype Redhawk did not produce any normal pods. In contrast, Sacramento, on average, produced ~ 25 plump seeds per plant. Plants produced fewer normal pods under elevated temperatures but far more parthenocarpic pin pods.

### Elevated temperatures increased leaf pigment concentrations

**Fig. 3:**
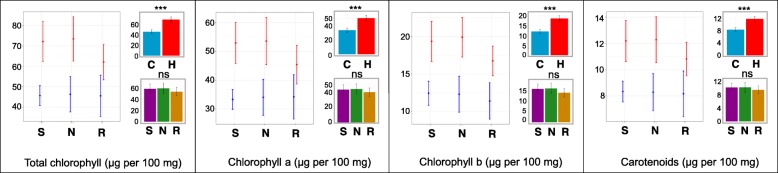
Leaf chlorophyll and carotenoid content in three bean genotypes (Sacramento, NY-105, and Redhawk) at flowering stage grown under control and heat stress condition. The amounts of chlorophyll and carotenoid were reported based on micro gram of pigments per 100 mg fresh weight of tissue. In each figure, the left graph represents the means for each genotype grown under control (blue) or heat (red) condition. The letters on each bar represents the results of post-hoc analysis. The same letter indicates the means are not significantly different at 0.05 probability level. The bars in all figures represent 95% confidence intervals. S = Sacramento, N = NY-105, R = Redhawk. C = control treatment, H = heat treatment

Total chlorophyll content, chlorophyll a, chlorophyll b, and carotenoids significantly increased in the elevated temperature treatment (Fig. 3). The increase in total chlorophyll content was greatest in Sacramento (58%) and NY-105 (59%). Redhawk increased chlorophyll content by only 36%.

### Elevated temperatures results in the accumulation of macro- and micro- nutrients in leaves

Among the 12 macro and micro- elements that were measured from the dried leaf tissues, 11 increased significantly under elevated temperature (Additional file 9: Figure S4). Potassium, sulfur, copper, and zinc were among the elements that were two-fold higher under elevated temperature. Aluminum was the only element that did not show any difference between the control and elevated temperature conditions.

### Sequencing, read alignment, and clustering of the samples

On average, about 1% of reads were discarded from libraries because they were low-quality (Additional file 10: Figure S5). About 34% of the reads did not align to the reference genome. The average number of aligned reads used for DEG analysis was ~ 18.5 million reads, with a range of 14.3–25.0 million reads per sample.

**Fig. 4:**
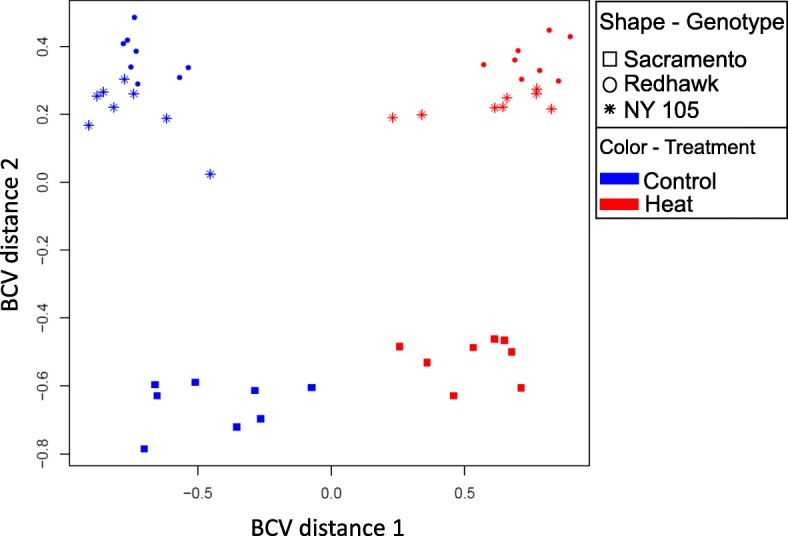
Unsupervised classification of samples based on their expression profiles. The samples from different levels of treatment (heat vs. control) were separated along the first dimension, indicating that the treatment was the main source of variation. The three genotypes were separated along the second dimension. Sacramento (square shape samples) was distantly separated from two other genotypes (NY-105 and Redhawk) along the second dimension

Unsupervised clustering of samples was performed based on expression profiles (Fig. 4). The samples belonging to the different treatments (control and elevated temperature) separated along the first dimension (BCV 1), while genotypes separated along the second dimension (BCV 2). Along the second dimension, Redhawk and NY-105 were clustered together while Sacramento individuals clustered distantly from those two genotypes.

### Identifying differentially expressed genes

**Fig. 5:**
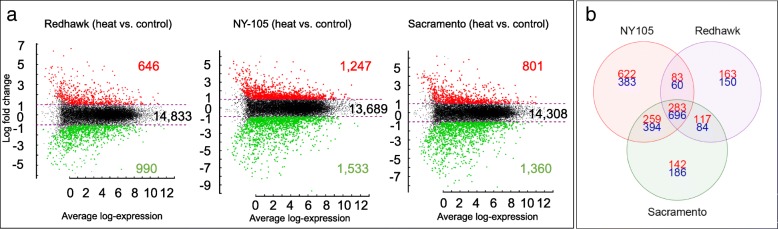
Differentially expressed genes under elevated temperatures in three varieties of common bean. **a** The overexpressed (red) and downregulated (green) genes under heat stress for three genotypes including Redhawk, NY-105, and Sacramento. **b** Venn diagram depicting the number of common up- or down- regulated genes under heat stress among genotypes

**Fig. 6:**
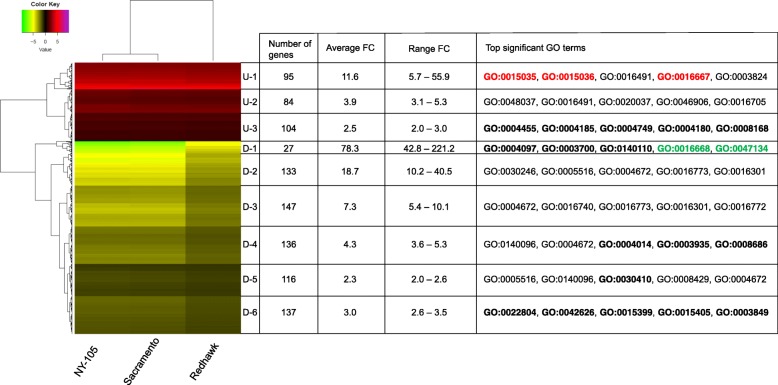
Heatmap indicating the log fold change of core genes expression under heat stress. Three and six clusters were identified within up- and down-regulated genes, respectively. Detailed information about clusters is provided in front of each cluster. GO terms uniquely detected in each cluster were bolded. GO terms related with protein disulfide regulations were highlighted by red in the U-1 cluster and green in the D-1 cluster

In total, 646, 1247, and 801 genes were upregulated under the elevated temperature treatment for Redhawk, NY-105, and Sacramento, respectively (Fig. 5a, Additional file 1). 990, 1533, and 1360 genes were downregulated in the elevated temperature treatment for Redhawk, NY-105, and Sacramento, respectively (Fig. 5a, Additional file 1). Among these, 283 genes were upregulated and 696 genes were downregulated under the elevated temperature among all three genotypes (Fig. 5b, Additional file 2). These genes, which responded in a similar way to the elevated temperature treatment across genotypes, are considered “core heat response genes”. Among some of the core genes were genes involved in carbohydrate and nitrogen metabolism, and genes contributing to thermotolerance and oxidative stress protection. Clustering analysis based on log-fold change of expression revealed three and six clusters of up- and down-regulated core genes, respectively (Fig. 6 and Additional file 3). The U-1 cluster contains the genes with the highest level of upregulation under heat stress for the three genotypes. In contrast, the D-1 cluster comprises the genes with the greatest level of down-regulation. Based on the expression profiles of all of the core genes, Sacramento and NY-105 were the most similar genotypes (Fig. 6). Further, 259 genes were upregulated and 394 genes were downregulated under elevated temperature in both of the tolerant genotypes (Sacramento and NY-105, Fig. 5b). We did not detect this set of genes among DEGs in Redhawk and consequently considered them as “tolerance-related heat response genes” (Additional file 4).

We further identified genes with significant genotype × treatment interactions. All three genotypes were fit in a single model and pairwise contrasts were conducted between all three genotypes. A total of 225 genes (Additional file 5) had significant genotype × treatment interactions for pairwise contrast between Sacramento and Redhawk. For the pairwise contrast between NY-105 and Redhawk, there were 484 genes with significant genotype × treatment interactions. The lowest number of genes (*n* = 99) with genotype × treatment interactions was detected for the Sacramento and NY-105 contrast.

### Gene ontology enrichment and pathway analyses

Gene Ontology (GO) enrichment was conducted separately for core genes (Table 1) and tolerance-related genes (Table 2). Results indicate that genes involved in oxidoreductase activity as well as heme and tetrapyrrole binding were significantly up-regulated under elevated temperature for all three genotypes (Table 1). The majority of down-regulated genes under elevated temperature had functions related to protein kinase activity, carbohydrate binding, and phosphotransferase activity. Three GO terms (GO:0015035, GO:0015036, and GO:0016667) were in the highest up-regulated cluster for core genes (U-1, Fig. 6). These GO terms are related to protein disulfide oxidoreductase activity. Interestingly, two GO terms (GO:0016668 and GO:0047134) were detected among the D-1 cluster, which is comprised of genes with the greatest degree of down-regulation. These terms are associated with protein-disulfide reductase activity.

**Table 1:** GO enrichment results for up-regulated and down-regulated genes under the elevated temperature treatment for core heat response genes

Down-regulated			Up-regulated		
GO	Term	*P*-value	GO	Term	*P*-value
GO:0004672	protein kinase activity	1.20E-08	GO:0016491	oxidoreductase activity	4.10E-07
GO:0030246	carbohydrate binding	7.00E-07	GO:0020037	heme binding	7.50E-07
GO:0016773	phosphotransferase activity, alcohol group as acceptor	8.30E-07	GO:0046906	tetrapyrrole binding	8.80E-07
GO:0016740	transferase activity	1.10E-06	GO:0016705	oxidoreductase activity, acting on paired donors	9.60E-07
GO:0016301	kinase activity	1.10E-06	GO:0048037	cofactor binding	4.80E-06
GO:0140096	catalytic activity, acting on a protein	4.60E-06	GO:0005506	iron ion binding	1.20E-05
GO:0005516	calmodulin binding	1.40E-05	GO:0015035	protein disulfide oxidoreductase activity	0.00015
GO:0016772	transferase activity, transferring phosphorus-containing groups	0.00012	GO:0003824	catalytic activity	0.00015
GO:0003824	catalytic activity	0.00024	GO:0015036	disulfide oxidoreductase activity	0.0002
GO:0016758	transferase activity, transferring hexosyl groups	0.00167	GO:0016667	oxidoreductase activity, acting on a sulfur group of donors	0.00049
GO:0016872	intramolecular lyase activity	0.00218	GO:0004553	hydrolase activity, hydrolyzing O-glycosyl compounds	0.00116
GO:0005524	ATP binding	0.00244	GO:0009055	electron transfer activity	0.00171
GO:0001871	pattern binding	0.00295	GO:0016798	hydrolase activity, acting on glycosyl bonds	0.00199
GO:0030247	polysaccharide binding	0.00295	GO:0046872	metal ion binding	0.00348
GO:0004970	ionotropic glutamate receptor activity	0.00346	GO:0043169	cation binding	0.00378
GO:0005230	extracellular ligand-gated ion channel activity	0.00346	GO:0046914	transition metal ion binding	0.00404
GO:0008066	glutamate receptor activity	0.00346	GO:0016161	beta-amylase activity	0.00435
GO:0015276	ligand-gated ion channel activity	0.00346	GO:0019139	cytokinin dehydrogenase activity	0.00511
GO:0022824	transmitter-gated ion channel activity	0.00346	GO:0008236	serine-type peptidase activity	0.0078
GO:0022834	ligand-gated channel activity	0.00346	GO:0017171	serine hydrolase activity	0.0078
GO:0022835	transmitter-gated channel activity	0.00346	GO:0016160	amylase activity	0.00975
GO:0030594	neurotransmitter receptor activity	0.00346	GO:0005088	Ras guanyl-nucleotide exchange factor activity	0.01198
GO:0008144	drug binding	0.00371	GO:0005089	Rho guanyl-nucleotide exchange factor activity	0.01198
GO:0016757	transferase activity, transferring glycosyl groups	0.00415	GO:0017048	Rho GTPase binding	0.01198
GO:0008429	phosphatidylethanolamine binding	0.0049	GO:0016702	oxidoreductase activity, acting on single donors	0.01388
GO:0004888	transmembrane signaling receptor activity	0.00504	GO:0070011	peptidase activity, acting on L-amino acid peptides	0.0141
GO:0038023	signaling receptor activity	0.00504	GO:0008233	peptidase activity	0.01719
GO:0060089	molecular transducer activity	0.00504	GO:0051213	dioxygenase activity	0.01937
GO:0032559	adenyl ribonucleotide binding	0.00585	GO:0016884	carbon-nitrogen ligase activity, with glutamine as amido-N-donor	0.0213
GO:0030554	adenyl nucleotide binding	0.00633	GO:0004455	ketol-acid reductoisomerase activity	0.0249
GO:0043565	sequence-specific DNA binding	0.00742	GO:0016645	oxidoreductase activity, acting on the CH-NH group of donors	0.02597
GO:0016705	oxidoreductase activity, acting on paired donors	0.01055	GO:0016701	oxidoreductase activity, acting on single donors with oxygen	0.0277
GO:0035639	purine ribonucleoside triphosphate binding	0.01262	GO:0016787	hydrolase activity	0.03003
GO:0016765	transferase activity, transferring alkyl or aryl (other than methyl) groups	0.01586	GO:0005085	guanyl-nucleotide exchange factor activity	0.04019
GO:0022839	ion gated channel activity	0.01729	GO:0004066	asparagine synthase (glutamine-hydrolyzing) activity	0.04116
GO:0022857	transmembrane transporter activity	0.02096	GO:0004749	ribose phosphate diphosphokinase activity	0.04116
GO:0032555	purine ribonucleotide binding	0.02266	GO:0005092	GDP-dissociation inhibitor activity	0.04919
GO:0022836	gated channel activity	0.02311	GO:0005094	Rho GDP-dissociation inhibitor activity	0.04919
GO:0005216	ion channel activity	0.02628	GO:0016778	diphosphotransferase activity	0.04919
GO:0022838	substrate-specific channel activity	0.02628			
GO:0032553	ribonucleotide binding	0.0265			
GO:0017076	purine nucleotide binding	0.02712			
GO:0015267	channel activity	0.03204			
GO:0022803	passive transmembrane transporter activity	0.03204			
GO:0050664	oxidoreductase activity, acting on NAD(P) H, oxygen as acceptor	0.0339			
GO:0097367	carbohydrate derivative binding	0.03397			
GO:0043168	anion binding	0.03531			
GO:0005506	iron ion binding	0.03867			
GO:0020037	heme binding	0.04013			
GO:0022804	active transmembrane transporter activity	0.0407			
GO:0000166	nucleotide binding	0.0408			
GO:1901265	nucleoside phosphate binding	0.0408			
GO:0016651	oxidoreductase activity, acting on NAD(P)H	0.0412			
GO:0005215	transporter activity	0.04162			
GO:0046906	tetrapyrrole binding	0.04373			
GO:0030410	nicotianamine synthase activity	0.04483			
GO:0005342	organic acid transmembrane transporter activity	0.04757			
GO:0015171	amino acid transmembrane transporter activity	0.04757			
GO:0046943	carboxylic acid transmembrane transporter activity	0.04757

**Table 2:** GO enrichment results for up-regulated and down-regulated genes under the elevated temperature treatment for tolerant-related heat response genes

Down-regulated			Up-regulated		
GO	Term	*P*-value	GO	Term	*P*-value
GO:0004672	protein kinase activity	4.00E-11	GO:0000175	3′-5′-exoribonuclease activity	0.011
GO:0016773	phosphotransferase activity, alcohol group as acceptor	7.90E-10	GO:0004532	exoribonuclease activity	0.011
GO:0016301	kinase activity	2.90E-09	GO:0004623	phospholipase A2 activity	0.011
GO:0140096	catalytic activity, acting on a protein	2.30E-08	GO:0016896	exoribonuclease activity, producing 5′-phosphomonoesters	0.011
GO:0016772	transferase activity, transferring phosphorus-containing groups	1.40E-07	GO:0003723	RNA binding	0.015
GO:0008061	chitin binding	4.60E-07	GO:0000295	adenine nucleotide transmembrane transporter activity	0.017
GO:0004568	chitinase activity	2.80E-05	GO:0004418	hydroxymethylbilane synthase activity	0.017
GO:0016740	transferase activity	0.00017	GO:0004654	polyribonucleotide nucleotidyltransferase activity	0.017
GO:0005199	structural constituent of cell wall	0.00258	GO:0004657	proline dehydrogenase activity	0.017
GO:0052736	beta-glucanase activity	0.00258	GO:0005346	purine ribonucleotide transmembrane transporter activity	0.017
GO:0052861	glucan endo-1,3-beta-glucanase activity, C-3 substituted reducing group	0.00258	GO:0005347	ATP transmembrane transporter activity	0.017
GO:0052862	glucan endo-1,4-beta-glucanase activity, C-3 substituted reducing group	0.00258	GO:0005471	ATP:ADP antiporter activity	0.017
GO:0003824	catalytic activity	0.00693	GO:0008839	4-hydroxy-tetrahydrodipicolinate reductase	0.017
GO:0008144	drug binding	0.00999	GO:0015215	nucleotide transmembrane transporter activity	0.017
GO:0003700	DNA-binding transcription factor activity	0.01435	GO:0015216	purine nucleotide transmembrane transporter activity	0.017
GO:0043565	sequence-specific DNA binding	0.01531	GO:0015217	ADP transmembrane transporter activity	0.017
GO:0140110	transcription regulator activity	0.02212	GO:0015301	anion:anion antiporter activity	0.017
GO:0005345	purine nucleobase transmembrane transporter activity	0.03958	GO:0015605	organophosphate ester transmembrane transporter activity	0.017
GO:0008198	ferrous iron binding	0.03958	GO:0016855	racemase and epimerase activity, acting on amino acids and derivatives	0.017
GO:0015205	nucleobase transmembrane transporter activity	0.03958	GO:0036361	racemase activity, acting on amino acids and derivatives	0.017
GO:0004185	serine-type carboxypeptidase activity	0.04819	GO:0005509	calcium ion binding	0.018
			GO:0016628	oxidoreductase activity, acting on the CH-CH group of donors	0.028
			GO:0016854	racemase and epimerase activity	0.028
			GO:0099516	ion antiporter activity	0.028
			GO:0008097	5S rRNA binding	0.033
			GO:0016796	exonuclease activity, active with either ribo- or deoxyribonucleic acids	0.039

Several pathways were screened for the presence of differentially expressed genes. Overall, protein kinases and genes involved in responses to cold and biotic stresses were down-regulated in the heat treatment (Additional file 11: Figure S6). In contrast, genes involved in heat stress, cell cycle regulation, and glutaredoxin activities were up-regulated.

### Sucrose, free hexoses, and starch measurement

**Fig. 7:**
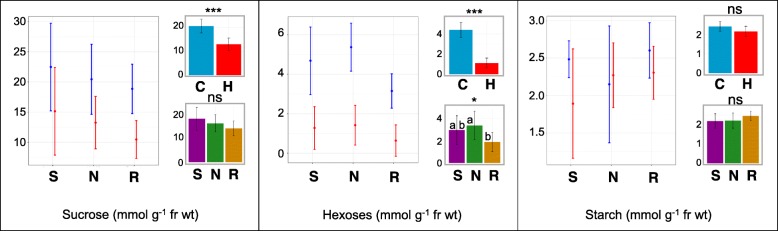
Leaf metabolite analysis of three bean genotypes (Sacramento, NY-105, and Redhawk) at flowering stage grown under control and heat stress condition. In each figure, the left graph represents the means for each genotype grown under control (blue) or heat (red) conditions. The upper right graph represents the main effect of treatments across genotypes and the graph below represents the main effect of genotypes across treatments. The letters on each bar represents the results of post-hoc analysis. The same letter indicates the means are not significantly different at 0.05 probability level. The bars in all figures represent the 95% confidence intervals. S = Sacramento, N = NY-105, R = Redhawk. C = control treatment, H = heat treatment

Elevated temperature significantly reduced the concentration of leaf sucrose (Fig. 7). Sucrose reduction was greatest (~ 2 fold) in the heat-susceptible genotype Redhawk. Furthermore, we detected a drastic reduction (~ 4 fold) in the concentration of free hexoses for plants under elevated temperature (Fig. 7). Among the genotypes, Redhawk had the lowest concentration of hexoses in both control and heat treatments. We did not detect any significant differences in starch content between the treatments.

### Correlations among morpho-physiological traits

To further investigate the relationships among morpho-physiological parameters, a correlation heatmap was constructed (Additional file 12: Figure S7). Based on this analysis, strong positive correlations were detected between photosynthetic rate and ΦPSII (r = 0.92, *P* < 0.001). A positive but weaker correlation (r = 0.49, *P* = 0.0004) was detected between the rate of photosynthesis and *g*_*s*_. Interestingly, no significant correlation was detected between the rate of photosynthesis and *C*_i_. Further, a strong correlation was detected between leaf area and stomatal density. This strong negative correlation (r = − 0.69, *P* < 0.001) between leaf area and stomatal density may indicate that leaf expansion results in decreasing the stomatal density.

## Discussion

In this study, we combined physiological and gene expression analyses to identify the mechanisms important for heat stress responses in common bean. Physiological measurements revealed that photosynthesis was not negatively impacted by the heat stress, which suggests that carbon assimilation is not the limiting factor in seed set under elevated temperatures. Instead, our transcriptome and physiological analyses indicate that crucial source-sink relationships are disrupted by the elevated temperatures. Based on the expression data, the control and elevated temperature samples clustered separately, indicating that the treatment was the main source of variation in this experiment. Below, we discuss the following three major topics regarding the effects of heat stress on physiology and gene regulation: *i*) effects of elevated temperature on leaf gas exchange and morpho-physiological characteristics, *ii*) potential effects of elevated temperature on source-sink relationships, and *iii*) detection of heat stress responsive transcription factors and other genes involved in heat tolerance.

### Elevated temperatures modified gas exchange and leaf morpho-physiology

In general, photosynthetic rate did not appear to be negatively affected by the heat stress imposed by our experiment. However, enhanced respiration rates during the early developmental stages indicate lower ratios of photosynthesis to respiration under elevated temperatures. Leaf area expansion can compensate for a lower ratio of photosynthesis to respiration and contribute to higher whole plant photosynthetic rates and daily carbon gains. We found that elevated temperature resulted in a drastic increase in leaf area, particularly for the most susceptible line, Redhawk. Redhawk also had the highest respiration rate at early vegetative stages. A higher ratio of dark respiration to photosynthesis at the onset of heat stress might result from growth respiration associated with the leaf area expansion.

At the flowering stage, a higher photosynthetic rate and ΦPSII was detected for plants under heat stress. ΦPSII is the proportion of absorbed light used in PSII photochemistry and reflects the rate of electron transport through PSII, which is equal to the combined rate of photosynthesis and photorespiration [13]. An increase in ΦPSII under moderate heat stress has been observed in previous studies and is attributed to high rates of photorespiration under heat stress [13].

We found higher stomatal conductance (*g*_*s*_) for plants under heat stress. By raising *g*_*s*_*,* a plant can decrease leaf temperatures via latent heat loss through evaporative cooling. Increased evaporative cooling resulting from increased *g*_*s*_ under heat stress was reported recently in *Pinus taeda* and *Populus deltoides* × *nigra* [37] as well as *Arabidopsis* [22]. We found that plants grown under elevated temperatures had a lower stomatal density. This finding is in accordance with a reduced stomatal density reported in *Arabidopsis* plants under heat stress [22]. The fact that stomatal conductance was higher, even though stomatal density was lower in plants grown under heat stress, indicates that stomata were more open in heat stressed plants. Crawford et al. [22] suggested that a reduction in stomatal density results in an increase in the inter-stomatal space and improves vapor diffusion. Another strategy that could potentially improve the cooling capacity of the plants is a decrease in leaf thickness, which has been observed in *Arabidopsis* [22]. Crawford et al. [22] argued that plants adopting these strategies could have enhanced cooling capacity under elevated temperature conditions.

### Potential effect of heat on source-sink relationships

Reproductive tissue functionality largely depends on carbon assimilation in the leaves and its subsequent delivery through the phloem to reproductive organs [38]. From a physiological standpoint, carbon allocation to reproductive tissues can be reduced by *i*) limitations in photosynthesis, *ii*) limitations in phloem loading at the source or unloading at the sinks, or *iii*) competition among other sinks for carbon. Regardless of the cause, limited carbon allocation to reproductive tissues results in aborted seed and/or reduced fruit set. Suboptimal environmental conditions, particularly drought [39], cold [40], and heat [18], are among the most devastating abiotic stresses that limit carbon allocations to flower buds, which results in drastic yield losses.

Although drought and heat both hinder carbon allocation to reproductive tissues, these stresses differ in their mechanisms of action. Drought hinders carbon allocation to reproductive tissues mainly by suppressing photosynthesis. Under water deficit, the net photosynthetic rate is typically significantly reduced while the respiration rate is affected less [41]. The lower rate of photosynthesis under drought seems to be a direct result of the ABA-dependent effect of stomatal closure [42]. However, moderate heat stress seems to limit the delivery of sucrose to sinks, rather than hindering photosynthesis [14]. In our experiment, there was a significant effect of heat stress on seed set. This is despite the fact that we could not detect negative effects of heat stress on biochemical or physiological aspects of photosynthesis. Therefore, under the levels of elevated temperatures tested in this study, seed set must be affected by factors other than changes in CO_2_ assimilation.

Changes in sugar and other nutrient transport from source leaves to developing pods and alterations in stress responsive transcription factors that specifically affect reproductive growth likely contribute to the deleterious effects of elevated temperatures. This is consistent with a study in maize, where heat did not affect the photosynthetic rate, but resulted in lower carbon allocation to the reproductive tissues [14]. Several studies have highlighted the important role of partitioning photosynthates under stress conditions [18, 43, 44]. In rice, drought and heat resulted in sugar starvation in multiple floral organs [43]. In tomato, lower starch accumulation occurred in pollen grains that had developed under heat stress [18]. Interestingly, in heat tolerant tomato cultivars, the starch accumulation in pollen grains was not affected by heat [44]. In our study, we found that sucrose transporter 2 (*SUT2*) expression was downregulated under elevated temperatures among all genotypes. *SUT* genes are major H^+^/sucrose symporters that play an important role in loading sucrose from leaves into the phloem as well as the subsequent translocation of sucrose into sinks [45]. We speculate that down-regulation of *SUT2* expression under elevated temperature is accompanied by lower export of carbon from source leaves to reproductive organs, which ultimately translates to yield reduction. Downregulation of sucrose transporter genes under heat stress was also reported in rice [46] and barely [47]. It was reported that *SUT* expression levels were significantly higher in heat tolerant rice genotypes compared with a susceptible line under elevated temperatures [48]. Under drought stress in maize [49] or heat stress in tomato [50], lower sucrose import was reported in the reproductive tissues, resulting in glucose depletion. This glucose depletion was sensed and triggered programed cell death (PCD), which resulted in the abortion of reproductive tissues [49, 50].

Other important gene families involved in carbon metabolism are *β*-amylases (BAM) and invertases. *β*-amylases catalyze starch breakdown to maltose [51, 52]. We detected increased expression of two *β*-amylase genes, *BAM3* and *BAM5*, under heat stress. BAM3 is localized in the chloroplast stroma of mesophyll cells [53]. BAM5 is a catalytically active cytosolic enzyme which was dominantly detected in phloem tissues [53, 54]. Higher activity of *β*-amylase by heat stress has been reported previously [55, 56]. Upregulation of *BAM3* and *BAM5* may result in increased maltose concentration. Kaplan and Guy [56] suggested that maltose serves as a compatible solute that protects the stromal proteins and the functionality of the photosynthetic electron transport chain under extreme temperatures.

Another key gene that was upregulated under elevated temperatures in our experiment is sucrose synthase 6 (*SUS6*). *SUS* family catalyze the reversible conversion of sucrose to nucleoside diphosphate-glucose and fructose. This reaction consequently results in the accumulation of starch. Thus, *SUS* family members are considered to be predominant regulators of carbon flow and are involved in both sink strength and phloem loading [57]. Considering the overexpression of the *SUS6*, *BAM3*, *BAM5*, as well as the downregulation of *SUT2*, it is plausible that under heat stress, carbon flow in the leaves shifts from sucrose export to starch synthesis and maltose formation.

Invertase genes are also involved in the regulation of carbon flow. Members of this family hydrolyze sucrose into hexoses and are classified into three main groups based on their patterns of expression within a plant [58]: cell-wall invertases (CWIN), vacuolar invertases (VIN), and cytosolic invertases (CIN). We found that the bean homolog to the *Arabidopsis CWIN1* gene exhibits reduced expression under elevated temperatures for all three genotypes. We observed a five-fold reduction of hexose concentration in the plants grown in the heat treatment. This might, at least partially, result from the lower expression levels of *CWIN1*. Previous studies have suggested that invertase activity and hexose concentrations have a negative feedback effect on photosynthetic rate [59] through sugar sensing mechanisms [60–62].

Beyond carbon allocation, nitrogen accumulation and the right carbon:nitrogen ratio is critical for seed set [63]. Two genes homologous to nitrate transporter 1:2 (*NRT1:2*) and one gene homologous to chloride channel B (*CLC-B*) were significantly upregulated under the elevated temperature treatment. These genes are involved in nitrate uptake [64, 65]. Furthermore, we detected higher expression of nitrite reductase (*NiR*) and glutamine synthetase N-1 (*GS*) under elevated temperatures. These two enzymes are localized in the chloroplast and play important roles in nitrogen assimilation [66]. A recent study found that overexpression of *NiR* results in higher chlorophyll content in tobacco leaves [67], which is consistent with the higher chlorophyll concentrations found in our experiment.

### Heat stress responsive transcription factors and genes involved in protection

Protein denaturation is one of the first and most drastic adverse effects of heat stress in biological systems. This can potentially affect several metabolic pathways by reducing the enzymatic activities. Higher presence of disulfide bonds in hyperthermophile organisms suggests the potential role of these bonds in the protein stability in hot environments [68]. In our experiment, we found that the highly up-regulated genes (cluster U-1, Fig. 6) are enriched for protein disulfide oxidoreductase activity. Interestingly the highly down-regulated genes (D-1, Fig. 6) are enriched for the protein-disulfide reductase function. These results indicate that enzymes involved in protein disulfide modifications shift their regulation dramatically under elevated temperatures. Although, upregulated genes showed the same levels of increase among genotypes, greater down-regulation levels were detected in tolerant genotypes particularly for the D-1 cluster (Fig. 6).

Heat shock factors (HSF) are among the most crucial transcription factors that orchestrate the physiological responses of plants to heat. Plants have developed a higher diversity of HSFs compared with animals. Although just four HSFs are found in animals [69], plant HSFs consist of multiple families with several members in each family [70]. Heat Shock transcription Factor A2 (*HsfA2*) is a critical gene induced under heat stress. Two genes homologous to *Arabidopsis HsfA2* were significantly overexpressed in our experiment under heat for all three genotypes. Loss of function mutations in this gene are associated with higher sensitivity to heat stress in *Arabidopsis* [71]. *HsfA2* regulates the expression of downstream genes encoding for chaperones and enzymes involved in heat tolerance [69]. Furthermore, *HsfA2* plays a role in histone methylation and epigenetic regulation for long-term acclimation to heat stress [72].

We detected a significant up-regulation of Growth-Regulating Factor 5 (*GRF5*) under heat stress for all three genotypes. *GRF5* is a transcription factor that has been shown to be involved in positive regulation of leaf growth, chloroplast division, and increased photosynthetic rate [73]. Overexpression of *GRF5* in *Arabidopsis* results in a significant increase in chloroplast number, without any detectible changes in chloroplast size, leaf thickness, or mesophyll cells size. These physiological modifications were associated with higher ETR, *q*_P_, CO_2_ assimilation, and WUA, particularly under higher light intensities (400 μmol photons m^− 2^, s^− 1^ or more). In our experiment, we detected the overexpression of *GRF5* along with higher chlorophyll content (Fig. 3) and photosynthetic rate under elevated temperatures (Fig. 1). Ectopic expression of *GRF5* is associated with overexpression of *PORA* (NADPH:Pchlide oxidoreductase A), which promotes chlorophyll synthesis and positive regulation of *GLK1*, a gene involved in chloroplast development. Expression of *PORA* and *GLK1* was significantly higher for Redhawk than the two tolerant genotypes.

Two homologues of the transcription factor SQUAMOSA PROMOTER BINDING PROTEIN-LIKE 12 (*SPL12*) were overexpressed under the elevated temperature treatment for all three genotypes. These genes are involved in thermotolerance and seed production in *Arabidopsis* and tobacco plants [74]. *SPL12* was shown to be expressed in both vegetative and inflorescence organs with a significantly higher expression levels in sepal and petals [74]. Double mutations of *spl1* and *spl12* resulted in sterility of plants under heat, due to partial failure in flower opening. Lower superoxide dismutase (SOD) and higher ROS accumulation was detected in the inflorescence of these double mutant plants.

Improving protective mechanisms to ameliorate the deleterious effects of oxidative stresses can contribute to better adaptation/acclimation of plants to heat stress. *HSP21* was upregulated for all three genotypes under heat stress. This gene is involved in chloroplast development under heat [75] and the encoded protein is principally involved in protection of PSII against oxidative stresses [76]. A homolog of the *Arabidopsis* gene abscisic acid-deficient 4 (*ABA4, AT1G67080.1*) was upregulated under heat in all three genotypes. *ABA4* codes for a protein involved in neoxanthin biosynthesis. Neoxanthin is the ultimate precursor of ABA and is also a carotenoid species that resides in the LHCII complex and protects PSII from photo-oxidative stresses [77]. A gene homologous to *AT2G40100.1,* which encodes for light harvesting complex photosystem II (*LHCB4.3*), was upregulated only in the two tolerant varieties under heat stress. Bianchi et al. [78] reported that this protein is a crucial component of PSII and is involved in structural integrity and photoprotection of this photosystem. Overexpression of *HSP21*, *ABA4*, *LHCB4.3* indicates a crucial role for PSII protection in plant survival under elevated temperatures.

Another gene family involved in the oxidative protection of plants is peroxidase family. Peroxidase family members possess diverse functions including involvement in tolerance to abiotic stresses [79]. We detected *Peroxidase 47* among upregulated genes in the tolerant genotypes under the elevated temperature condition. This gene was upregulated ~ 5-fold in Sacramento and NY-105, but was not detected among DEG in Redhawk. This gene might be involved in the protection of tolerant genotypes against oxidative stresses.

## Conclusions

Overall, our study suggests that mechanisms other than photosynthesis play a primary role in intraspecific genetic variation in heat tolerance in common bean. For example, we found evidence that lower expression of leaf sucrose transporters (*SUT2*) at elevated temperatures may limit the transport of photosynthates from source to sinks and consequently starve the developing pollen and newly fertilized seeds. Further, carbon and nitrogen metabolism is likely significantly altered under elevated temperatures, given that associated genes were often differentially expressed in the heat treatment. The lack of heat damage to photosynthetic apparatus could potentially be explained by genes involved in photoprotection of photosystem II, which were upregulated under elevated temperatures. We also found that genes involved in protein disulfide modifications shift dramatically in their regulation in response to heat, which could contribute to higher protein stability and consequently enzymatic functionality under the heat stress.

Besides shedding new light on the transcriptome and physiological aspects of heat stress, the results of our study suggests potential trajectory for breeding for heat-tolerance. The fact that we could not find any relationship between photosynthesis rate and the heat tolerance indicate that selection for high photosynthesis under elevated temperatures in breeding programs is unlikely to be efficient way to develop heat tolerant beans. Instead, more emphasis should be given to other traits, especially those involved in source-sink relationships. Although we could not find any relationship between photosynthesis rate and heat tolerance, we cannot rule out the effect of photosynthesis completely. Variation in the mechanisms of heat tolerance could exist in common bean germplasm and thus, evaluation of more diverse genotypes will be necessary. Considering the multidimensionality of heat stress and the mechanisms of tolerance, an integrative approach should be employed in the future studies. To identify the pivotal genetic factors involved in heat tolerance mechanisms, it will be necessary to conduct genetic mapping through Quantitative Trait Locus and/or Genome-Wide Association Studies. Measuring physiological and metabolite parameters in combination with transcriptome and proteome data from several tissues, including source and sink, will provide crucial information about the mechanisms of heat tolerance. Finally, designing experiments to track changes in carbon and nitrogen allocation from sources to sinks should provide insights about nutrient partitioning during heat stress.

## Methods

### Plant materials

Prior to conducting a detailed physiological and gene expression analysis, we screened *P. vulgaris* germplasm to identify the most tolerant genotypes at elevated temperatures. We selected lines for screening based on observations of heat tolerance in the field for these lines made by Dr. Timothy Porch (USDA-ARS; unpublished data). The goal of this pilot experiment was to confirm heat tolerance under growth chamber conditions and select the two most heat tolerant genotypes for further experimentation. All plants were grown under the heat and control conditions, as described in the growth conditions section below. The genotypes in this initial experiment included four candidate heat tolerant genotypes: Sacramento (light red kidney), NY-105 (light red kidney), G122 (cranberry), and TARS HT1 (dark red kidney), as well as two candidate susceptible genotypes: Camelot (dark red kidney) and Lisa (white kidney). Dr. Phillip Miklas (USDA-ARS WA) and Dr. Timothy Porch (USDA-ARS PR) provided these seeds. Four replicates of each genotype were grown in heat and control conditions. Genotypes were randomized within each treatment. Four traits including the number of filled pods, number of pin pods, total number of seeds and seed weight were measured to evaluate the fitness of these genotypes under elevated temperature (Additional file 6: Figure S1). Two criteria were considered for choosing the most tolerant genotypes for downstream experiments; *i*) seed and pod set were similar across control and heat stress conditions, indicating tolerance and *ii*) low variability for each genotype in each treatment (Additional file 6: Figure S1), reflecting higher stability of critical traits. Sacramento and NY-105 were the most heat tolerant varieties with the lowest amount of variation in this initial experiment due to their higher seed set under the elevated temperature conditions. Following this pilot experiment, we set up the main experiment with the two most heat tolerant genotypes and a heat susceptible genotype, Redhawk (dark red kidney). Redhawk was selected because it is the primary line being grown commercially for agricultural production of kidney beans. Dr. James Kelly (Michigan State University) provided the seeds of Redhawk.

### Experimental design

Sacramento, NY-105, and Redhawk genotypes were germinated under 29/20 °C (day and night temperatures, respectively) in two identical growth chambers (Big foot, Biochambers, Winnipeg, MB, Canada). Plants were grown in 3.79 L pots filled with 2 SUREMIX (SURE, Galesburg, MI, USA):1/2 sand. Eight replicates of each genotype were grown in each chamber with individual plant position randomized within each chamber. Upon reaching the V4 developmental stage, heat treatment plants were exposed to 32/25 °C until physiological maturity. Note, we use the terms “heat” and “elevated temperature” interchangeably to describe the high temperature treatment. In contrast, the control plants were kept at 29/20 °C throughout the duration of the experiment. The control and heat temperatures were selected based on the previous studies showing that bean production is limited by day and night time temperatures above 30 °C and 20 °C, respectively [28]. Plants were frequently watered to avoid any confounding effects of water stress. The photoperiod was set for 16-h days and 8-h nights. For both treatments, the LED light intensity (25% blue, 75% red) was set to 500 μmol photons m^− 2^ s^− 1^ and the relative humidity set to 60%.

The experiment was conducted as a completely randomized design using eight replicates per treatment and genotype. The plants were rearranged every week within each growth chamber to minimize the effect of location. For each trait, the differences between means was tested by performing ANOVAs in the R environment using the aov() function, followed by post-hoc Tukey tests. The Pearson correlations among physiological traits were calculated and plotted in R using the psych and corrplot packages.

### Photosynthetic parameters

A LI-6800 portable gas exchange system (LI-COR Biosciences, Lincoln, NE) connected to a Multiphase Flash™ Fluorometer (6800-01A) was used to obtain gas exchange and chlorophyll *a* fluorescence measurements simultaneously. Photosynthetic rates (*A*), stomatal conductance (*g*_*s*_), operational efficiency of photosystem II in light adapted leaves (ΦPSII), and pre-dawn respiration rates were quantified in this way.

Daytime gas exchange measurements were made during the 3rd to the 8th hr. of the photoperiod. Predawn respiration rates were measured within two hr. prior to the beginning of the photoperiod. The LI-6800 was set up outside the growth chamber and the following conditions were maintained in the LI-6800 leaf chamber to reflect daytime growth chamber conditions: leaf temperature of 29 °C (control), 32 °C (heat stress); sample [CO_2_] of 400 μmol mol^− 1^; 500 μmol m^− 2^ s^− 1^ light intensity (25% blue, 75% red); leaf vapor pressure deficit of 1.5–2.0 kPa. During predawn respiration measurements, leaf chamber lights were switched off and the leaf temperature was set to 20 °C (control) and 25 °C (heat stress). To prevent circadian effects on gas exchange measurements, selection of plants for measurements were alternated between the control and the heat stress chambers. A Fluke infrared thermometer (Model 68, Fluke Corporation, Washington, USA) was used to determine the actual surface temperature of the leaves while plants were inside of the growth chambers. These measurements were taken from the same leaves that were used for gas exchange measurements. Leaf surface temperatures were measured during the day, between the 3rd and 8th hr. of the photoperiod, and also during the nighttime within the two hours prior to the beginning of the photoperiod.

### Chlorophyll and carotenoid content

Leaves were ground to a fine powder using mortar and pestle, following flash freezing with liquid nitrogen. One hundred mg of frozen tissue was weighted and transferred to each tube. One ml of 95% ethanol was added to each sample and thoroughly mixed. The tubes were then centrifuged at 17000 g at 4 °C for 5 min. The supernatant for each sample was transferred to a fresh tube and diluted by the addition of 1 ml of 95% ethanol. The absorbance of samples was measured by GENESYS 10S UV-Vis spectrophotometer (ThermoFisher, Waltham, MA) at three wavelengths: 470 nm, 649 nm, and 665 nm. The amount of chlorophyll a (C_a_), chlorophyll b (C_b_) and carotenoids (C_c_) were estimated using the following equations [80]:

1 $$ {\mathrm{C}}_{\mathrm{a}}=13.95{A}_{665-}\;6.88{A}_{649} $$

2 $$ {\mathrm{C}}_{\mathrm{b}}=24.96{A}_{649}-7.32{A}_{665} $$

3 $$ {\mathrm{C}}_{\mathrm{c}}=\frac{l000{A}_{470}-2.05{C}_a-114.8{\mathrm{C}}_{\mathrm{b}}}{245} $$

### Leaf area and relative water content

Leaf area was quantified for the left leaflet of the fourth trifoliate using the LI-3100 Area Meter (LI-COR INC, Lincoln, NE). Fresh weight (FW) of the same leaflet was measured simultaneously. The leaflet was then placed in a plastic bag containing a saturated paper towel. After 24 h, the turgid weight (TW) of each leaf was measured. The leaves were then placed in a drying oven, set to 60 °C, for five days, at which point the dry weight (DW) was measured. Relative water content (%) was calculated using eq. 4:

4 $$ RWC\ \left(\%\right)=\frac{FW- DW}{TW- DW}\times 100 $$

### Stomatal density

Leaf surface imprints were acquired from the abaxial and adaxial surfaces of the terminal leaflet of the fourth trifoliate leaf. Digital photographs of imprints were used to quantify stomatal density using ImageJ 1.51 [81].

### Leaf macro- and micro- nutrient measurement

Twelve macro and micro nutrients were measured from the left leaflet of the 4th trifoliate leaf at the Brookside Laboratories, Inc., New Bremen, OH. Tissues were dried at 60 °C overnight and ground using a Cyclotech Mill with a 0.5 mm screen. Total nitrogen was measured by a combustion method. To measure the minerals, tissues were digested by nitric acid/hydrogen peroxide in conjunction with microwave heating in closed Teflon vessels. Samples were then analyzed by a Thermo iCAP 6500 spectrometer (Thermo Scientific, Waltham, MA).

### RNA extraction, library preparation and sequencing

Total RNA was extracted from the inner side (the side closer to the terminal leaflet) of the left lateral leaflet of the fourth trifoliate using Spectrum™ Plant Total RNA Kit (Sigma-Aldrich, St. Louis, MO). The quantity and quality of extracted RNA was evaluated using Qubit fluorometer (Invitrogen INC. Carlsbad, CA) and bioanalyzer 2100 (Agilent Technologies, Santa Clara, CA), respectively. Library preparation and sequencing were performed at the RTSF Genomics Core at Michigan State University. Libraries were prepared using the Illumina TruSeq Stranded mRNA Library Preparation Kit. Sequencing was performed on an Illumina HiSeq 4000 flow cell in the 1x50bp single end read configuration.

### Cleaning and mapping the reads

FastQC (v 0.11.3) was used to assess the quality of raw reads and identify the potential overrepresented sequences (adaptors) in each sequencing file. Adaptor sequences were trimmed with Cutadapt (v 1.14) and the quality of reads was confirmed by running additional analyses with FastQC (v 0.11.3). Cleaned reads were then aligned to the *P. vulgaris* reference genome (v 2.1) using HISAT2 (v 2.1.0, [82]). Htseq-count [83] was used to count the number of reads for each feature of *P. vulgaris.* The *P. vulgaris* reference genome and annotated GFF3 files were downloaded from Phytozome (v 12.1).

### Detecting differentially expressed genes (DEG), GO enrichment and pathway analysis

The read count data were imported into R [84] and genes with count per million (cpm) ≥1 in at least eight samples were retained in the analysis. The differentially expressed genes were identified using the limma package [85] after voom transformation [86]. The voom function was used to estimate the relationship between the mean and variance of log-counts. Consequently, a precision weight is estimated and assigned to each observation. After this normalization step, data are ready for normal linear modelling. Log-fold-change of 1 or − 1 was considered the threshold for differentially expressed genes. We used the “global” method of the decideTests function to identify significant DEG. The gene ontology enrichment was performed using Fisher’s exact tests with the topGO package [87]. Pathway analysis and corresponding visualizations were performed by MapMan 3.6.0 [88].

### Measuring soluble sugars

The amount of sucrose, glucose, and fructose were measured from the right leaflet of the fourth trifoliate leaf. Leaf samples were taken between two to three hours after the onset of the photoperiod. Briefly, 300 mg of ground tissue was mixed with 3.5% perchloric acid. Following centrifugation, the supernatant containing the soluble sugars was separated from the pellet, which contained the starch fraction. The supernatant was neutralized using a buffer containing 2 M KOH, 150 mM Hepes, and 10 mM KCl. Five microliters of each sample were then transferred into wells of a 96-well plate. We then transferred a buffer containing 110 mM Hepes, 500 nmol NADP, 500 nmol ATP, and 0.4 units of glucose-6-phosphate dehydrogenase (Sigma G-8529) to each well. The baseline optical density (OD) at 340 nm was measured by an MDS M2 plate reader. One unit of hexokinase (Sigma, I-4504) and 50 units of invertase (Sigma, H-4502) were the added to each well. The concentration of samples was calculated using 6220 M^− 1^ cm^− 1^ for the extinction coefficient of NADPH.

### Measuring starch

The starch pellet from the previous section was washed three times with 80% ethanol. The pellet was then dried for 5 min at 95 °C. Following desiccation, 0.2 M KOH was added to gelatinize the pellet. The samples were incubated at 95 °C for 30 min. The pH was adjusted to 5 by adding 1 M acetic acid. Starch was degraded by adding 6.6 units of amyloglucosidse and 50 units of α-amylase. Ten μl of supernatant was then transferred to each well and absorption levels were recorded using the same protocol as explained in the previous section.

## Additional files

Additional file 1: List of up- and down-regulated genes under heat stress condition in each of three genotypes of common bean. (XLSX 535 kb)

Additional file 2: List of core genes that up- and down-regulated in all three genotypes. (XLSX 111 kb)

Additional file 3: List of core genes in the same order as the heatmap in Fig. 6. (XLSX 60 kb)

Additional file 4: List of genes that differentially expressed under the heat stress conditions and just detected in tolerant genotypes (Sacramento and NY-105). (XLSX 1518 kb)

Additional file 5: List of genes showing genotype × treatment interaction. (XLSX 88 kb)

Additional file 6: **Figure S1.** Effect of heat on seed set traits for six bean genotypes (Camelot, Sacramento, NY-105, G-122, TARS-HT1 and Lisa) screened in the pilot experiment. The means for each genotype is indicated by blue (control) and red (heat stress). The bars in all figures represent the 95% confidence intervals. (PDF 63 kb)

Additional file 7: **Figure S2.** Leaf stomatal density measured from abaxial and adaxial surface of three bean genotypes (Sacramento, NY-105, and Redhawk) at flowering stage grown under control and heat stress condition. In each figure, the left graph represents the means for each genotype grown under control (blue) or heat (red) conditions. The upper right graph represents the main effect of treatments across genotypes and the graph below represents the main effect of genotypes across treatments. The letters on each bar represents the results of post-hoc analysis. The same letter indicates the means are not significantly different at 0.05 probability level. The bars in all figures represent the 95% confidence intervals. S = Sacramento, N = NY-105, R = Redhawk. C = control treatment, H = heat treatment. (PDF 36 kb)

Additional file 8: **Figure S3.** Effect of heat on reproduction of three bean genotypes (Sacramento, NY-105, and Redhawk). In each figure, the left graph represents the means for each genotype grown under control (blue) or heat (red) conditions. The upper right graph represents the main effect of treatment across genotypes and the graph below represents the main effect of genotypes across treatments. The letters on each bar represents the results of post-hoc analysis. The same letter indicates the means are not significantly different at 0.05 probability level. The bars in all figures represent the 95% confidence intervals. S = Sacramento, N = NY-105, R = Redhawk. C = control treatment, H = heat treatment. (PDF 61 kb)

Additional file 9: **Figure S4.** Leaf macro- and micro-nutrient content of three bean genotypes (Sacramento, NY-105, and Redhawk) grown under control and heat stress condition. In each figure, the left graph represents the means for each genotype grown under control (blue) or heat (red) conditions. The upper right graph represents the main effect of treatments across genotypes and the graph below represents the main effect of genotypes across treatments. The letters on each bar represents the results of post-hoc analysis. The same letter indicates the means are not significantly different at 0.05 probability level. The bars in all figures represent the 95% confidence intervals. S = Sacramento, N = NY-105, R = Redhawk. C = control treatment, H = heat treatment. (PDF 178 kb)

Additional file 10: **Figure S5.** Summary of read numbers for each of the 48 libraries sequenced for the RNA-seq gene expression analysis. (PDF 3 kb)

Additional file 11: **Figure S6.** Overall regulation overview (upper row) and cellular response overview (lower row) of differentially expressed genes in three bean genotypes; Sacramento, NY-105 and Redhawk under heat stress. Red and blue colors indicate up- and down-regulation of genes, respectively. (PDF 366 kb)

Additional file 12: **Figure S7.** Correlation heatmap among 11 physiological and metabolite parameters. Positive and negative correlations indicated by blue and red, respectively. LA = leaf area, A = Photosynthesis rate, RES = respiration rate, Gs = stomatal conductance, Ci = internal [CO_2_], PII = operational efficiency of photosystem II in light adapted leaves (ΦPSII), STM = stomatal density in leaf abaxial, Hex = concentration of free hexoses, Suc = sucrose concentration, Seed = number of seeds per plant, and Chl = total chlorophyll concentration. (PDF 6 kb)
