# Supplementary figures and images for: Elevated temperatures cause loss of seed set in common bean (Phaseolus vulgaris L.) potentially through the disruption of source-sink relationships
Source: BMC Genomics. 2019 Apr 24;20:312. doi: 10.1186/s12864-019-5669-2 (PMC6480737; doi:10.1186/s12864-019-5669-2)

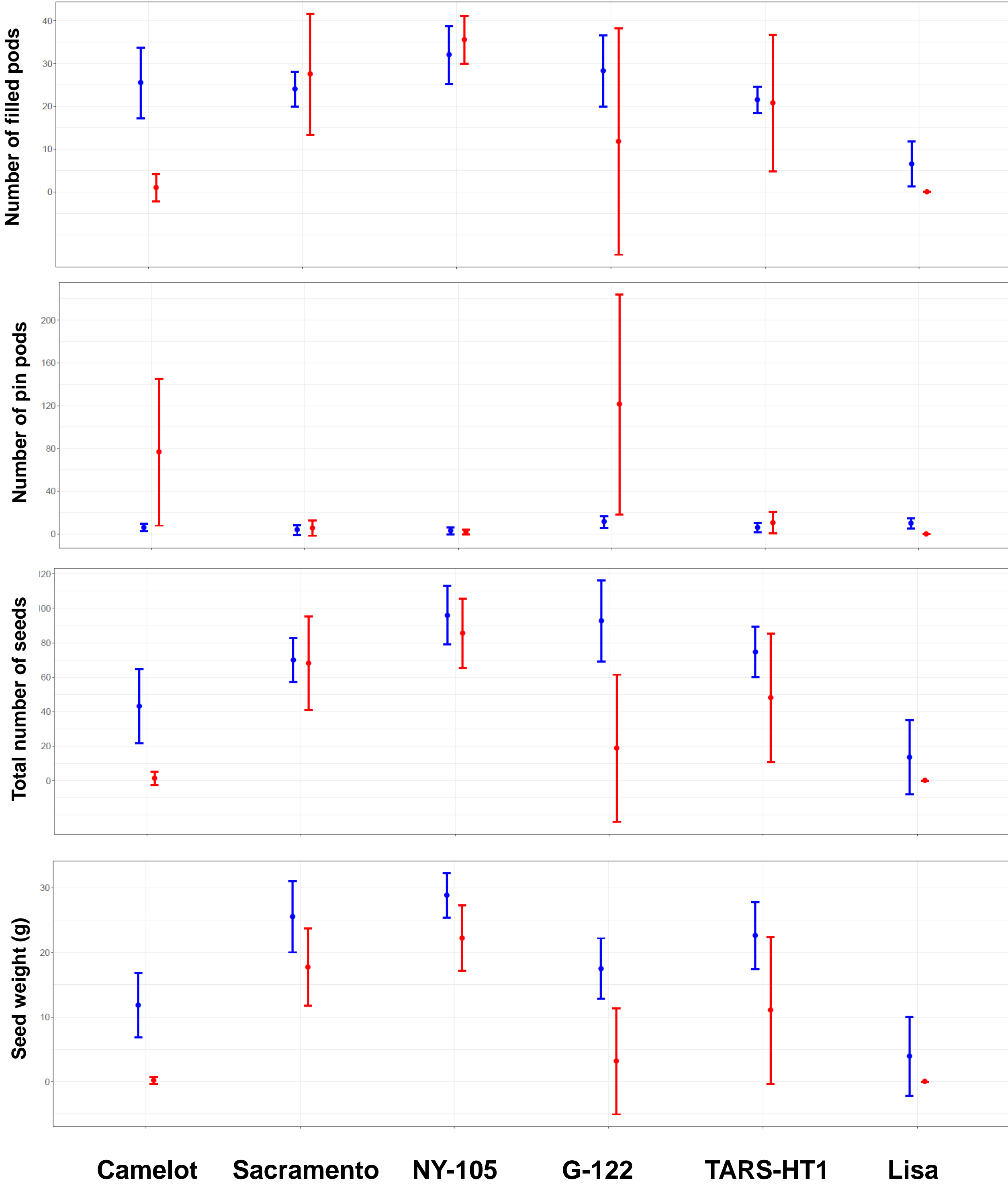

Supplement: Supplementary file 6 — Figure S1. Effect of heat on seed set traits for six bean genotypes (Camelot, Sacramento, NY-105, G-122, TARS-HT1 and Lisa) screened in the pilot experiment. The means for each genotype is indicated by blue (control) and red (heat stress). The bars in all figures represent the 95% confidence intervals. (PDF 63 kb) [file 12864_2019_5669_MOESM6_ESM.pdf]

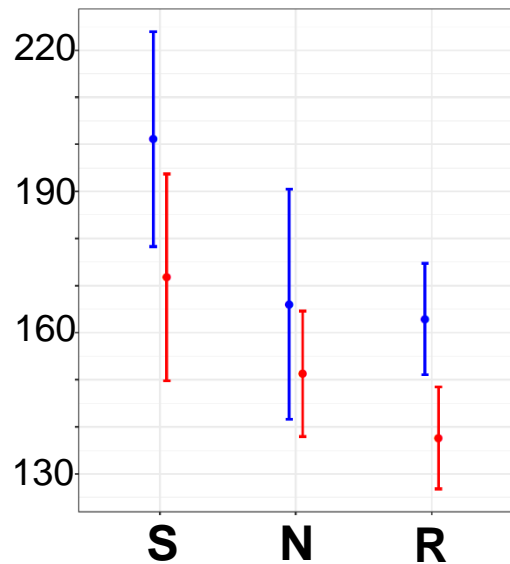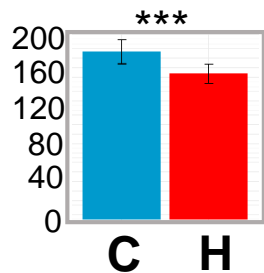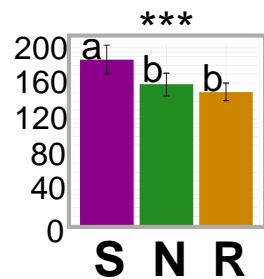

Leaf abaxial stomatal density

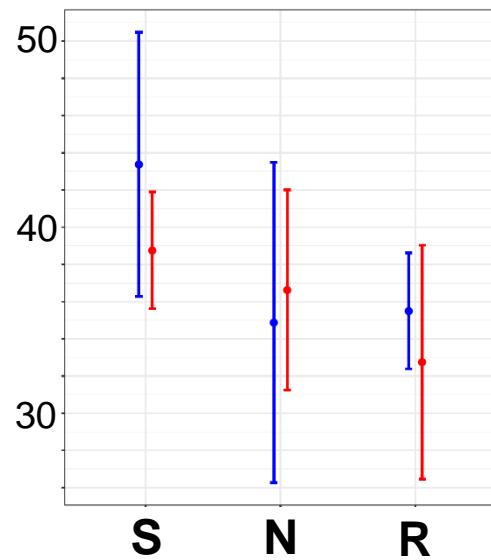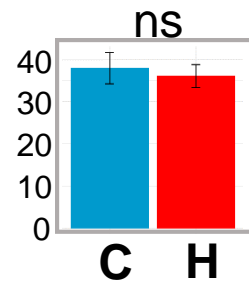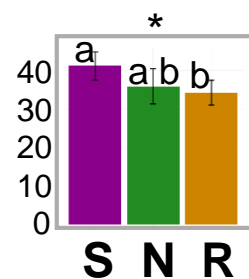

Leaf adaxial stomatal density

Supplement: Supplementary file 7 — Figure S2. Leaf stomatal density measured from abaxial and adaxial surface of three bean genotypes (Sacramento, NY-105, and Redhawk) at flowering stage grown under control and heat stress condition. In each figure, the left graph represents the means for each genotype grown under control (blue) or heat (red) conditions. The upper right graph represents the main effect of treatments across genotypes and the graph below represents the main effect of genotypes across treatments. The letters on each bar represents the results of post-hoc analysis. The same letter indicates the means are not significantly different at 0.05 probability level. The bars in all figures represent the 95% confidence intervals. S = Sacramento, N = NY-105, R = Redhawk. C = control treatment, H = heat treatment. (PDF 36 kb) [file 12864_2019_5669_MOESM7_ESM.pdf]

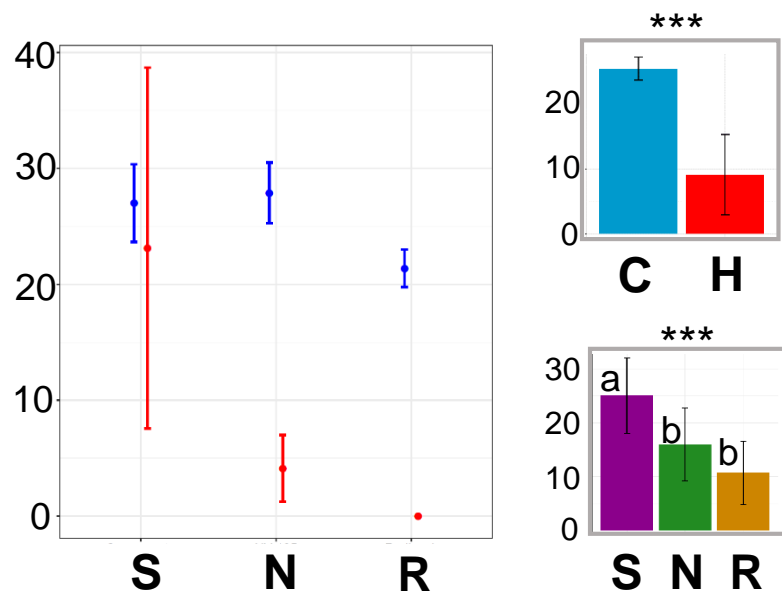

Filled pods

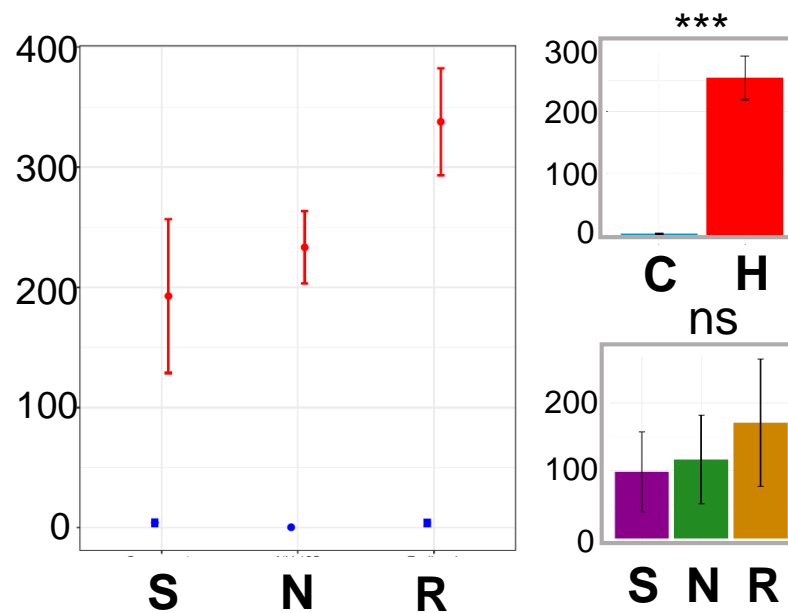

Pin pods

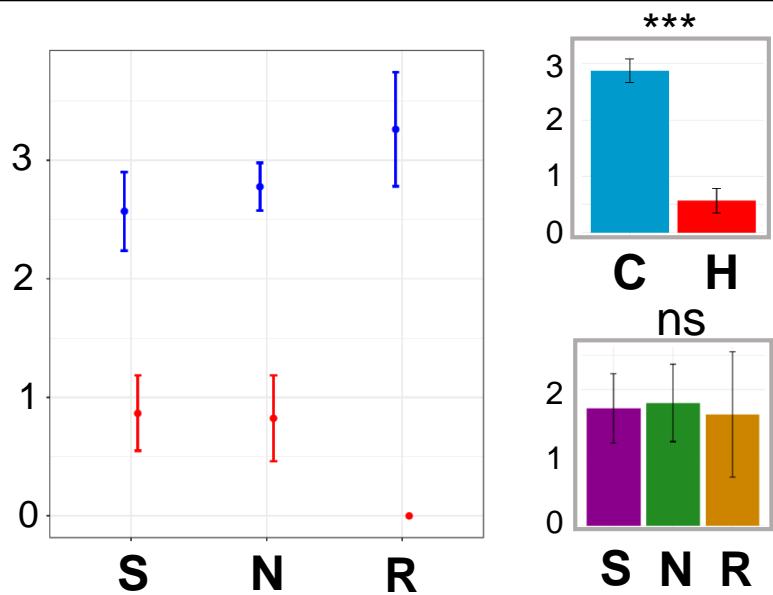

Seed per pod

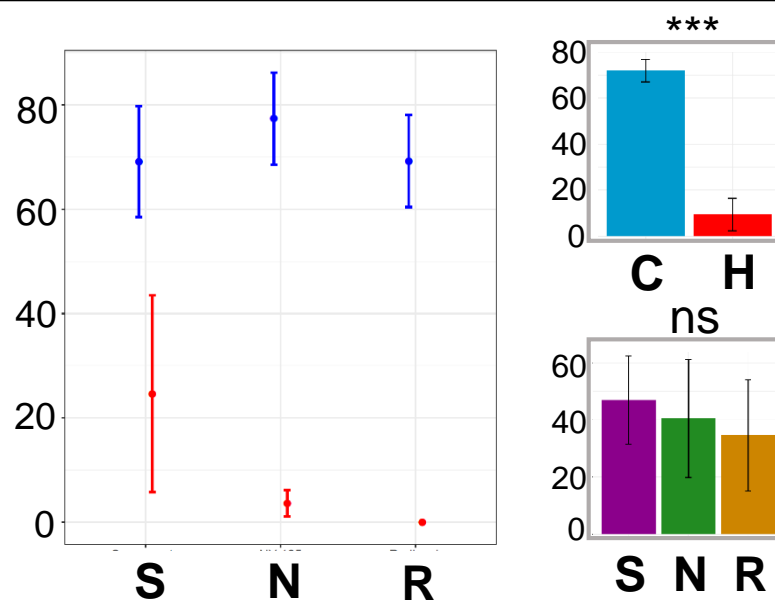

Plump seeds

Supplement: Supplementary file 8 — Figure S3. Effect of heat on reproduction of three bean genotypes (Sacramento, NY-105, and Redhawk). In each figure, the left graph represents the means for each genotype grown under control (blue) or heat (red) conditions. The upper right graph represents the main effect of treatment across genotypes and the graph below represents the main effect of genotypes across treatments. The letters on each bar represents the results of post-hoc analysis. The same letter indicates the means are not significantly different at 0.05 probability level. The bars in all figures represent the 95% confidence intervals. S = Sacramento, N = NY-105, R = Redhawk. C = control treatment, H = heat treatment. (PDF 61 kb) [file 12864_2019_5669_MOESM8_ESM.pdf]

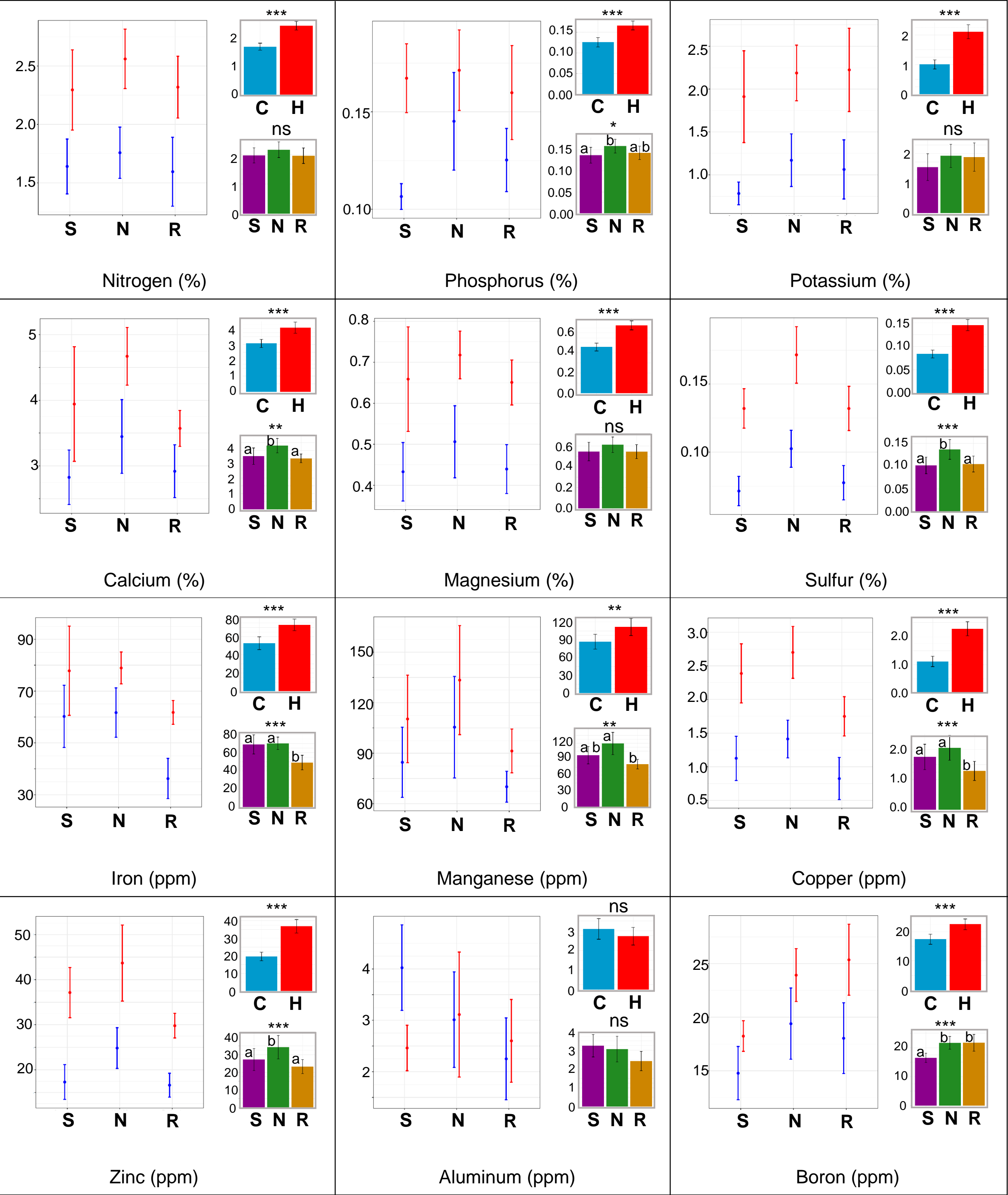

Supplement: Supplementary file 9 — Figure S4. Leaf macro- and micro-nutrient content of three bean genotypes (Sacramento, NY-105, and Redhawk) grown under control and heat stress condition. In each figure, the left graph represents the means for each genotype grown under control (blue) or heat (red) conditions. The upper right graph represents the main effect of treatments across genotypes and the graph below represents the main effect of genotypes across treatments. The letters on each bar represents the results of post-hoc analysis. The same letter indicates the means are not significantly different at 0.05 probability level. The bars in all figures represent the 95% confidence intervals. S = Sacramento, N = NY-105, R = Redhawk. C = control treatment, H = heat treatment. (PDF 178 kb) [file 12864_2019_5669_MOESM9_ESM.pdf]

Library sizes

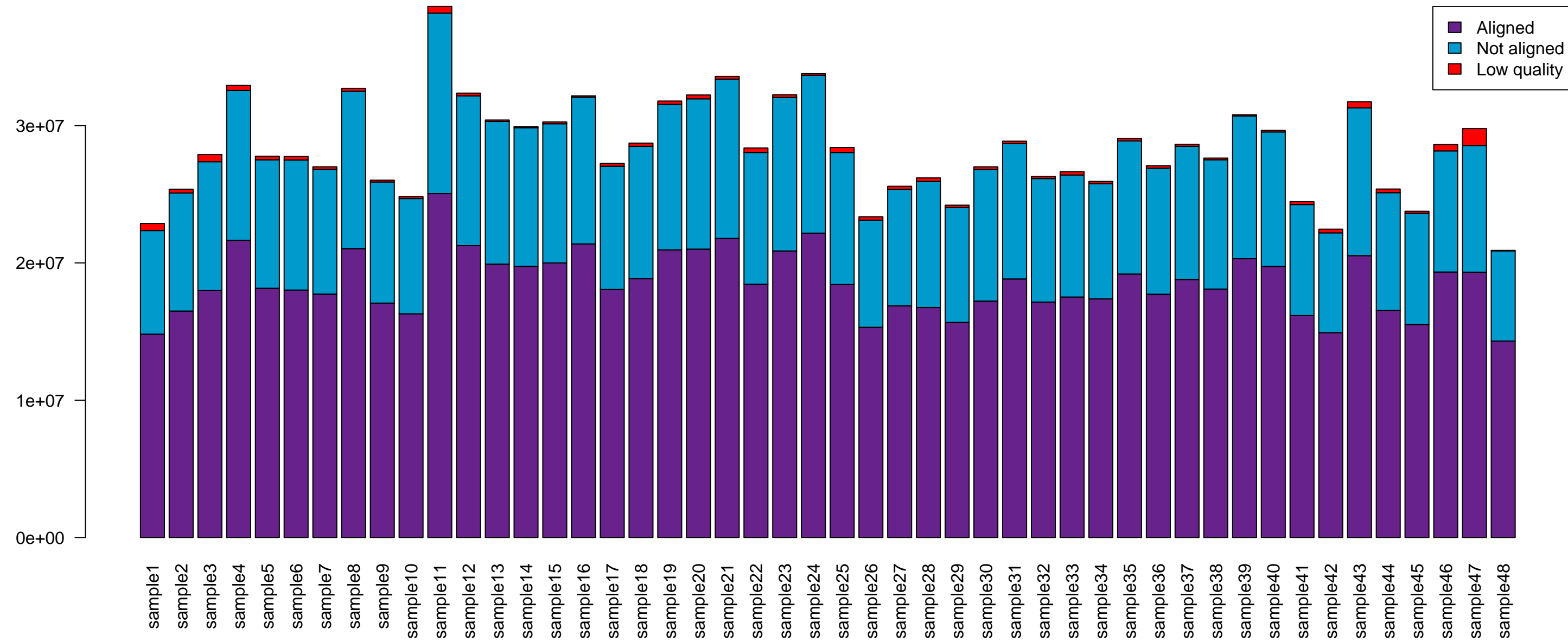

Supplement: Supplementary file 10 — Figure S5. Summary of read numbers for each of the 48 libraries sequenced for the RNA-seq gene expression analysis. (PDF 3 kb) [file 12864_2019_5669_MOESM10_ESM.pdf]

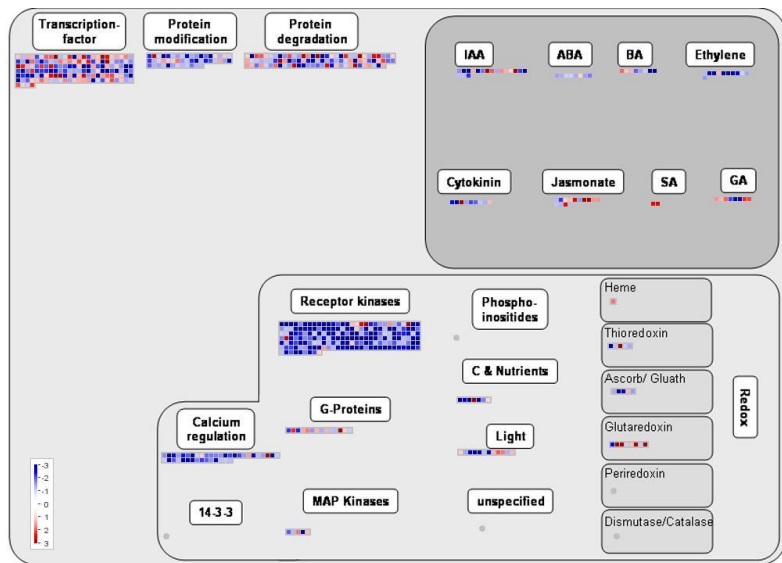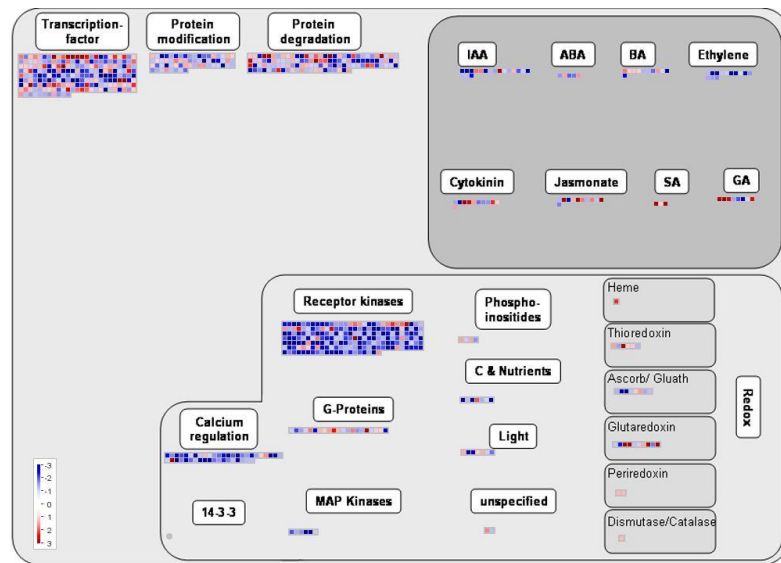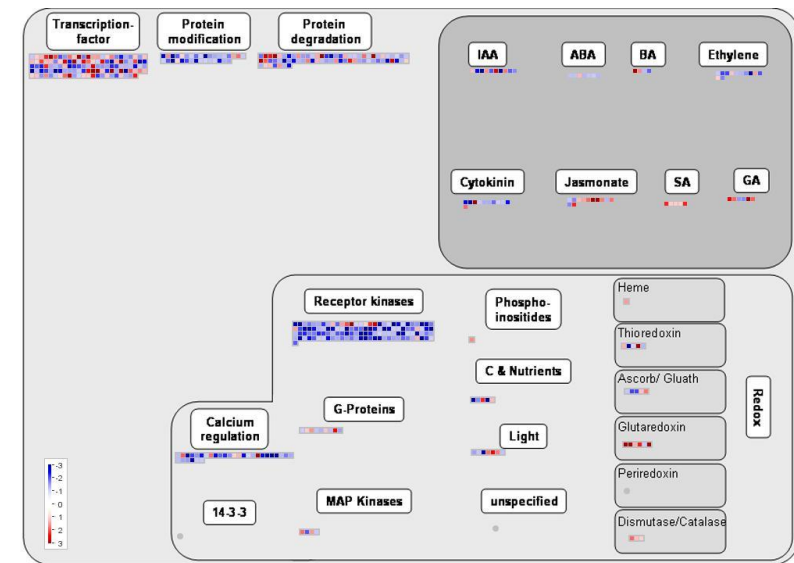

Sacramento

NY-105

Redhawk

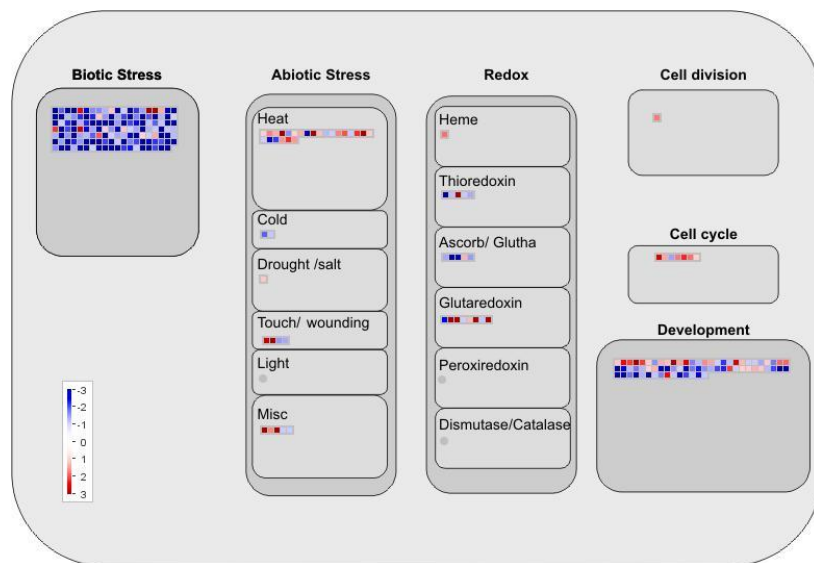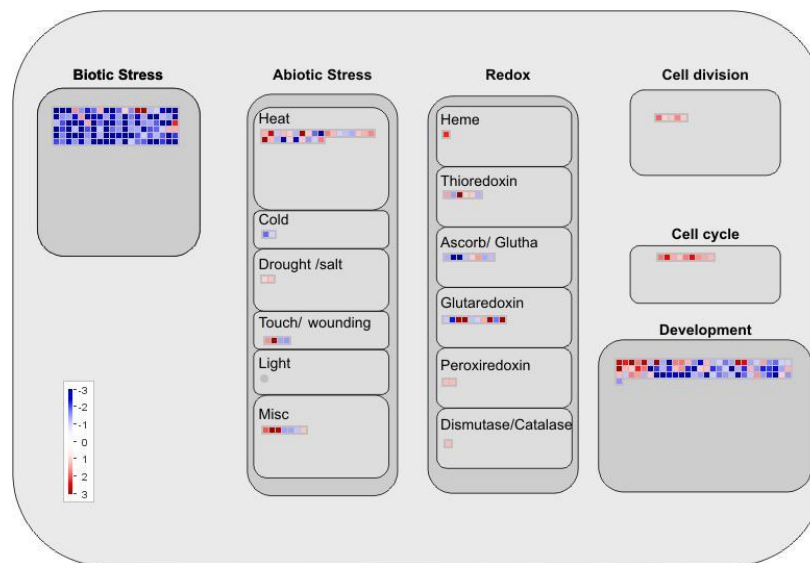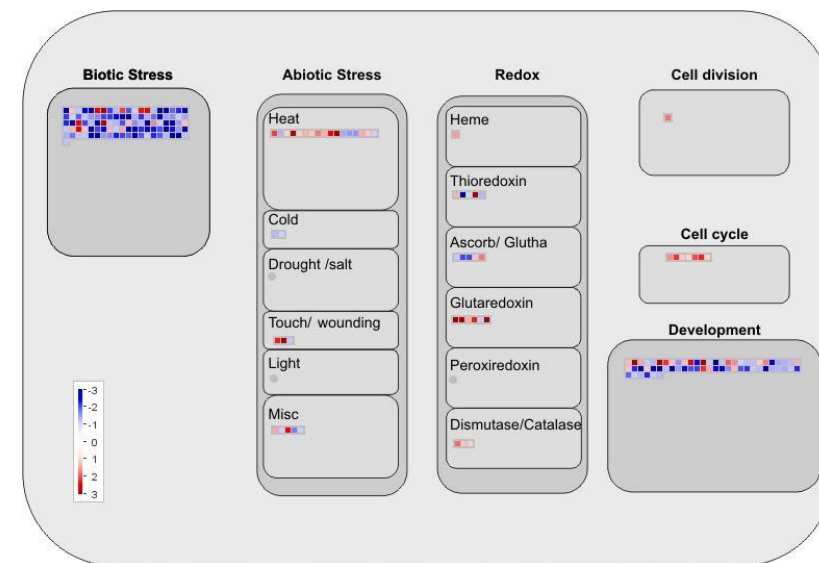

Supplement: Supplementary file 11 — Figure S6. Overall regulation overview (upper row) and cellular response overview (lower row) of differentially expressed genes in three bean genotypes; Sacramento, NY-105 and Redhawk under heat stress. Red and blue colors indicate up- and down-regulation of genes, respectively. (PDF 366 kb) [file 12864_2019_5669_MOESM11_ESM.pdf]

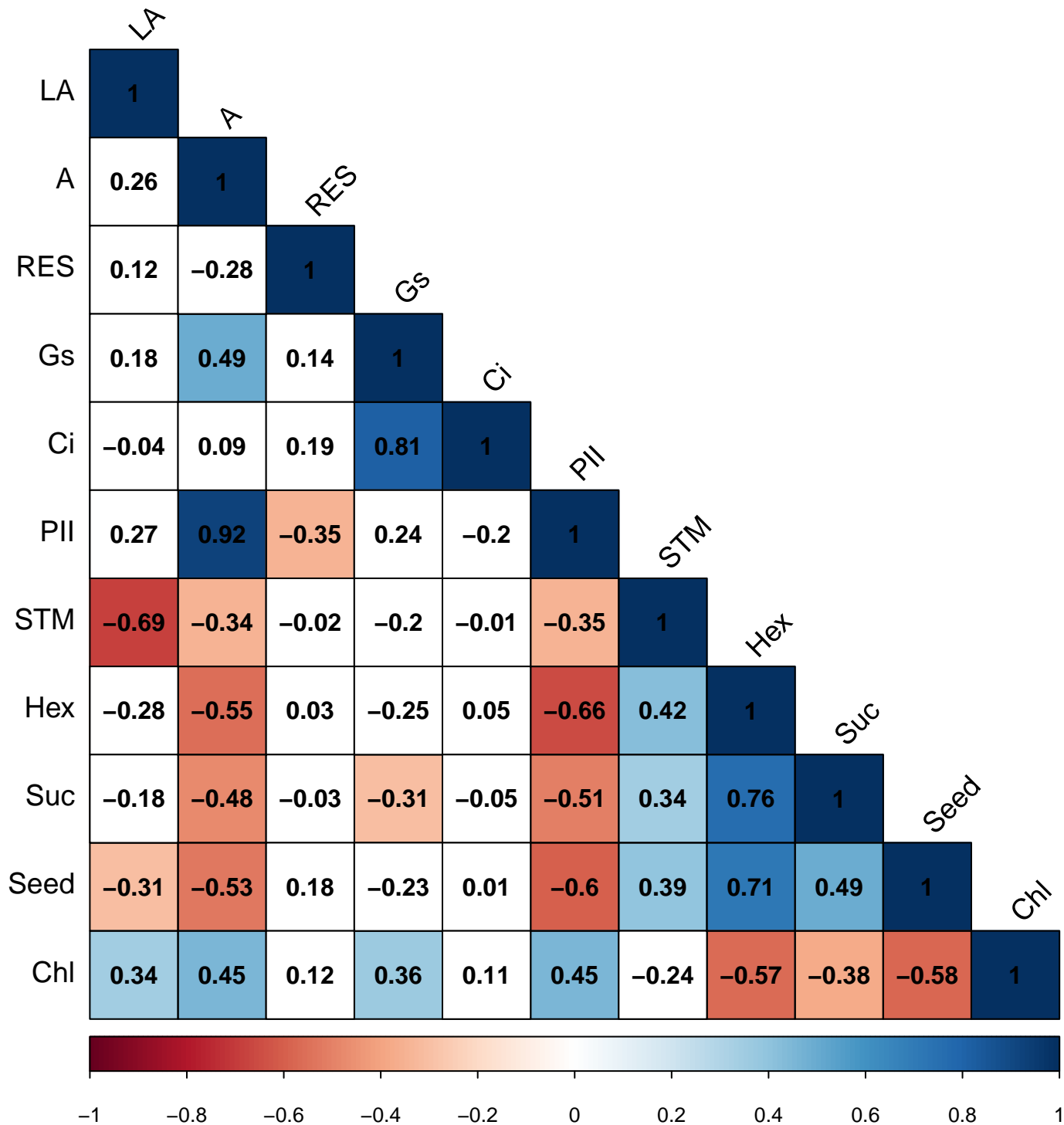

Supplement: Supplementary file 12 — Figure S7. Correlation heatmap among 11 physiological and metabolite parameters. Positive and negative correlations indicated by blue and red, respectively. LA = leaf area, A = Photosynthesis rate, RES = respiration rate, Gs = stomatal conductance, Ci = internal [CO2], PII = operational efficiency of photosystem II in light adapted leaves (ΦPSII), STM = stomatal density in leaf abaxial, Hex = concentration of free hexoses, Suc = sucrose concentration, Seed = number of seeds per plant, and Chl = total chlorophyll concentration. (PDF 6 kb) [file 12864_2019_5669_MOESM12_ESM.pdf]
